# Gap Dynamics and Structure of Two Old-Growth Beech Forest Remnants in Slovenia

**DOI:** 10.1371/journal.pone.0052641

**Published:** 2013-01-07

**Authors:** Tihomir Rugani, Jurij Diaci, David Hladnik

**Affiliations:** Biotechnical Faculty, Department of Forestry and Renewable Forest Resources, University of Ljubljana, Ljubljana, Slovenia; DOE Pacific Northwest National Laboratory, United States of America

## Abstract

**Context:**

Due to a long history of intensive forest exploitation, few European beech (*Fagus sylvatica* L.) old-growth forests have been preserved in Europe.

**Material and Methods:**

We studied two beech forest reserves in southern Slovenia. We examined the structural characteristics of the two forest reserves based on data from sample plots and complete inventory obtained from four previous forest management plans. To gain a better understanding of disturbance dynamics, we used aerial imagery to study the characteristics of canopy gaps over an 11-year period in the Kopa forest reserve and a 20-year period in the Gorjanci forest reserve.

**Results:**

The results suggest that these forests are structurally heterogeneous over small spatial scales. Gap size analysis showed that gaps smaller than 500 m^2^ are the dominant driving force of stand development. The percentage of forest area in canopy gaps ranged from 3.2 to 4.5% in the Kopa forest reserve and from 9.1 to 10.6% in the Gorjanci forest reserve. These forests exhibit relatively high annual rates of coverage by newly established (0.15 and 0.25%) and closed (0.08 and 0.16%) canopy gaps. New gap formation is dependant on senescent trees located throughout the reserve.

**Conclusion:**

We conclude that these stands are not even-sized, but rather unevenly structured. This is due to the fact that the disturbance regime is characterized by low intensity, small-scale disturbances.

## Introduction

Virgin and old-growth forests are important ecosystem cornerstones. Although they are well represented in the world (35.7% of forest area), only 2.8% of Europe's (Russian Federation excluded) forest area is classified as primary forest [1]. Human land use has a long history in Europe, where almost no areas have been left to retain their natural, unaltered character [2], [3]. The importance of understanding such ecosystems has been emphasized by several researchers [4], [5], [6].

European beech (*Fagus sylvatica* L.) is one of the most common of the many autochthonic tree species in Europe. Its range extends from Western Europe to the Carpathians in the east, and from Scandinavia in the north to Greece in the south, and is able to thrive in a broad range of soil and climate conditions from the lowlands to the timberline. European beech is a strong competitor and is thus present in a variety of mixed species forests as well as in pure stands [7]. These characteristics make it a dominant tree species in Central Europe. Because of the growing trend towards ecologically-based forest management, an understanding of the natural patterns and processes in beech stands is essential to help inform management practices. A number of studies have been carried out in the few remaining beech old-growth stands in Northern [8], [9], Western [10], Central [11], [12], [13], [14], [15], [16], Eastern [17], and Southern [18], [11], [19] Europe. Despite this body of work, there is still debate concerning the natural dynamics of beech stands. A common idea is that beech forms homogeneous stands dominated by large canopy trees with a sparse understory and middle layer, often referred to as ‘cathedral-like’ stands [2]. Two conceptual models have been proposed to explain this structure. One model suggests that after stand replacing events a new wave of even-aged regeneration occupies the canopy [2]. A second model suggests that a ‘cathedral-like’ stand can develop because beech grows rapidly through the middle height layer when light is available (i.e. in gaps), thereby resulting in an uneven-aged stand that has a uniform height structure and a diverse diameter structure (i.e. a wide range of diameters exist in the canopy layer) [13]. In contrast, a third model may suggest that beech stands have a heterogeneous age and size structure at small to intermediate spatial scales depending on the particular disturbance history of the stand, whereby recruitment to the upper layers of the stand is driven by spatiotemporal variation in light availability.

Many studies carried out in old-growth beech stands in Europe have used traditional approaches that focus on structural characteristics, such as descriptions of size distributions and mapping of development phases [6]. Until recently, few studies in the region have used methods that examine the processes behind the observed patterns. In temperate forests worldwide, numerous studies have shown the influence of gap disturbance processes on forest dynamics [20], [21], [22], [23], [24], [25]. Similarly, recent work in old-growth beech forests in Europe has provided valuable insight into natural disturbance processes for this forest type [11], [14], [16], [26]. In this study, we use time series data on forest structure and canopy gaps to investigate how two old-growth beech forest remnants in Slovenia fit the three previously mentioned a conceptual model, which has not explicitly been addressed in past work.

## Materials and Methods

### 1. Ethics statement

All necessary permits were obtained for the described field studies. The permits were issued by the Forestry service of Slovenia (Central unit). Gorjanci forest reserve is privately owned and the permit was issued by the owner's legal representative (Roman Zaletelj).

### 2. Study area

We studied two beech-dominated old-growth forest reserves: the Gorjanci and Kopa reserves. Both reserves are located in the northern part of the Dinaric mountain range, which lies in Slovenia.

The Gorjanci forest reserve is situated on the ridge of the Gorjenci massif, on the northern slope (45°45′N, 15°19′E). The ridge top (Trdinov vrh) is 1178 m a.s.l. and lies on the border between Slovenia and Croatia. The reserve encompasses 22.98 ha and ranges from 990 to 1150 m a.s.l. According to the nearest (1.6 km) meteorological station (Sv. Miklavz, 969 m a.s.l.), it has a continental climate with a mean annual precipitation of 1290 mm and a mean temperature of 12°C from March to September. The topography is steep (8–30°) with localized flat areas. The geology is comprised predominately of dolomite bedrock, but limestone is also locally present [27]. The vegetation is dominated by European beech (*Fagus sylvatica* L.), while other species, such as sycamore maple (*Acer pseudoplatanus* L.) and silver fir (*Abies Alba* Mill.), are less frequent. Large beech trees are present throughout the entire reserve but do not exceed 115 cm in diameter at breast height (d.b.h.) and 45 m in height. The reserve was not managed after World War Two and is considered not to have had any human interventions for at least a century. The forest reserve had not been accessible until a forest road was built to Trdinov vrh after World War Two. During our survey we did not find any remains of cut stumps.

The Kopa forest reserve is situated under the ridge top (Kopa, 1077 m a.s.l.) of the Kocevski Rog massif and has a northeast aspect (45°37′N, 15°02′E). Its size is 14.05 ha, and it ranges from 980 to 1080 m a.s.l. The climate is continental with a mean annual precipitation of 1240 mm and a mean temperature of 12.1°C from March to September, according to the nearest (5.1 km) meteorological station (Planina – Mirna gora, 740 m a.s.l.). The topography is moderately sloped and is characterized by Karst phenomena (e.g. sinkholes). The geology is comprised of limestone with substantial surface rockiness [27]. The vegetation is dominated by beech, while other species, such as sycamore maple, European ash (*Fraxinus excelsior* L.), and silver fir, are less frequent (<10% in the growing stock). The reserve has not been managed for more than 50 years. No cut stumps or other indications of human influence have been found.

### 3. Field sampling and data analysis

We examined the structural characteristics of the two forest reserves based on data from 24 (Gorjanci) and 21 (Kopa) circular sample plots with a radius of 15 m (i.e., 700 m^2^ in size) established on a systematic grid (100×100 m) and measured in 2004 and 2006, respectively. We measured the d.b.h. (1.30 m) of all (live and dead) trees (d.b.h.≥10 cm in Gorjanci and d.b.h.≥5 cm in Kopa), the position of all trees (live and dead), and the height of the three closest trees to the centre of the sample plots. We sampled lying (mean diameter was measured) and standing (d.b.h. was measured) dead trees. In Slovenia the monitoring of forest reserves is conducted in the framework of forest inventories. A drawback is that the circular sample plots used in the forest inventory are too small to draw conclusions on large-scale variation based on the pair correlation function for exploratory data analysis and the commonly used Ripley's K-function in spatial statistics [28], [29]. The spatial characteristics of forest stands are often estimated on the basis of evaluating the neighbourhood of a given number of reference trees [30]. Our aim was to assess the spatial distribution of trees at a scale comparable with that of small canopy tree fall. As the usefulness of the nearest neighbour distances for spatial pattern analysis is very limited [31], we analysed the spatial distribution of trees in the sample plots by calculating the distances and variation of these distances from the centre of the plot to all trees in the plot, a method based on the work of [32] and [33] and also presented and tested in [34]. This analysis follows the [35] index as a measure of the departure from randomness in a population of plants [36]. presented the mathematical derivations for a random point process (i.e. homogeneous Poisson process) as an extension of the index to the next nearest neighbours to detect larger-scale heterogeneity by only using the distance to the nearest neighbour.

We calculated average distances Rk to the k-th nearest trees from plot centers and compared them with theoretical distances according to [36]:
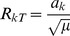
(1)where a_k_ is a constant, calculated for particular tree values up to *k* = 15 or the minimum number of trees on the sampling plots:
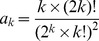
(2)and μ is density, calculated from tree number (N_ha_/10 000). Based on the second moment of the mean distance to the nearest trees presented by Thompson, the theoretical variation of distances was calculated:
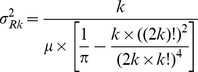
(3)It was shown by [37] that the actual coefficient of variation depends on the average values of distances between trees and the variance of these differences, whereas the theoretical coefficient of variation, which is calculated for a random distribution of trees, is dependent only on the *k* value, which represents the sequence of distances:
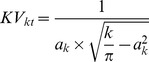
(4)On this basis we may conclude that the coefficient of variation of the distances between trees can be used as an indicator of the spatial distribution of trees. As shown in [34], the distances and variation of these distances on sampling plots can be compared with theoretical values, derived from [36].

We calculated the coefficients of variation of the distances from the centers of the plots to all trees (d.b.h.≥5 cm in Kopa and d.b.h.≥10 cm in Gorjanci), and of dominant trees (trees in the upper canopy layer), and compared them with a theoretical coefficient for random spatial distribution. If the variation of distances from the plot center to the k-tree is positioned above the theoretical line, the distribution of trees shows a tendency towards clustered distribution. If the variation of distances from the plot center to the k-tree is positioned below the theoretical line, the distribution of trees shows a tendency towards uniform distribution. Dominant trees in sample plots were estimated as a sample of the 100 largest (based on diameter) trees per hectare; in 700 m^2^ circular sample plots this would require the seven largest trees per plot. The average height of the 100 largest trees per hectare (i.e., quadratic mean diameter (*d_q_*_100_), defined as the diameter corresponding to the mean basal area of the 100 largest trees per hectare) is often used in Europe as an estimate of stand top height (h_dom_) [38], [39]. Based on the measurements in the sampling plots, we calculated the quadratic mean diameter and the regression height of the largest tree sample in both forest reserves (Gorjanci (*d_q_*_100_ = 63.8 cm; h_dom_ = 31.9 m) and Kopa (*d_q_*_100_ = 64.0 cm; h_dom_ = 34.0 m)). The canopy layer was estimated to exceed 2/3 of the dominant height (22 m) and the middle layer was estimated to exceed 1/3 of the dominant height (11 m). Lower layer trees were all trees under 11 meters in height.

To better understand the structural characteristics and their dynamics, we analyzed the complete inventory data (full callipering of all trees with d.b.h≥10 cm) obtained from the four previous forest management plans for the two management units [40], [41]. All live trees (d.b.h.≥10 cm) were measured at approximate 10-year intervals (4 measurements over a period of 31 years in Kopa and 35 years in Gorjanci) in both forest reserves.

To gain a better understanding of the disturbance dynamics, we studied the characteristics of the canopy gap disturbance regime. The predominant disturbance agent in the observed forest reserves was wind, which resulted in stem breakage or uprooting (personal observation). A canopy gap was delineated if regeneration in the gap was shorter than half (20 m) of the upper canopy height. Gaps were delineated from aerial photographs using a digital stereo plotter (Summit Evolution, DAT/EM Systems) for precise delineation of canopy gaps. Digital aerial images from the cyclic aerial survey of Slovenia were used for the delineation of gaps in 2006. The older panchromatic black-and-white (PAN) and Kodak 2443 color infrared (CIR) aerial film photographs were scanned using 21 µm resolution to achieve comparable resolution (Table 1). During the analysis the stereoscopic pairs of aerial photographs were interpreted and compared with terrain work based on the absolute orientation of stereoscopic photographs which established the reference to the terrain coordinate system. The absolute orientation of the stereopairs was based on at least 10 to 15 ground control points. The residuals of the X and Y coordinates for each point were allowed to be less than 1 m. The root mean square error (RMSE*xyz*) for the stereo model of the Gorjanci forest reserve was 0.36 m and 1.14 m for the Kopa reserve. The delineated gaps were compared with the corresponding ground truth, based on GPS positions of individual gaps. For the 22 gaps in the Gorjanci forest reserve we determined the position of the gaps with a sub-meter GPS (Trimble GeoXT) with differential correction. GPS use within the forest reserve was hindered by canopy cover and terrain, which blocked satellite signals from reaching the GPS receiver, so it was not possible to record the positions of single trees accurately. Capturing data in forest gaps that were clear of direct over-head cover improved the results of post-processed, differentially corrected data (average errors were between 2 and 3 m). In the field we measured the distances from the GPS position within forest gaps to the crown outline of the closest canopy tree. These distances were compared in the GIS environment with the corresponding distances of delineated tree crowns in the stereo model. For 22 ground GPS points the average difference to the crown positions on the delineated gaps was 2.47 m with standard error of 0.42 m.

**Table 1:** Summary of the characteristics of the image data used.

Study area	Acquisition date	Mapping scale	Focal length	Spectral resolution	Sensor
Gorjanci	17.7.2006	1∶20 000*	120 mm	CIR	Z/I DMC
	2/9/1986	1∶5 000	305 mm	CIR	Zeiss LMK 1000
Kopa	21.7.2006	1∶44 000*	120 mm	CIR	Z/I DMC
	18.7.1998	1∶17 500	210 mm	PAN	Zeiss LMK 1000

(*) Mapping scale estimate based on the digital mapping camera (DMC) from Z/I Imaging with 12 µm pixel size and 25 cm to 50 cm ground sample distance (GSD). For 2009 the UltraCamXp Vexcel Imaging camera was used (orthoimages with GSD of 25 cm).

We delineated gaps larger than 20 m^2^ (five meters in diameter), which could have been the result of a small canopy tree fall. This was a methodological constraint since it is difficult to delineate small forest gaps from aerial photographs of shaded hillsides, as it is not possible to clearly distinguish between regeneration in the gap and the trees of the upper canopy height. Trees in small gaps may be shaded or partially hidden by others, and the illumination conditions in the smaller gaps confounded efforts to delineate them successfully using visual stereo-image interpretation. Similar problems of misclassification of the non-dominant tree species in image segmentation and semi-automatic tree species classification approaches have been reported elsewhere [42]. We analyzed a 20-year time series of aerial photographs for the Gorjanci reserve (1986 and 2006) and an 11-year series (1998 and 2009) for the Kopa reserve. This data were used to quantify gap size distribution, gap fraction, and gap dynamics processes. We quantified new gap formation (newly formed gaps in the observed period), gap expansion (gaps that were expanded in the observed period), gap reduction (gaps that decreased in size in the observed period), and gap closure (gaps that closed in the observed period) in the GIS environment (ArcGIS). For each of these four gap dynamic processes, we calculated the number and area of gaps. The annual proportion of the total area covered by new gaps was calculated:
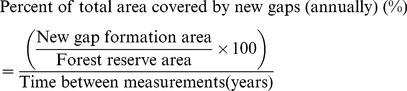
(5)The annual proportion of the total area covered by closed gaps was calculated:
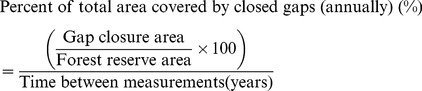
(6)We also carried out a more detailed analysis of new gap formation in which we studied the relationship between newly formed gaps and the presence of gaps in the first measurement. Newly established gaps were classified into several groups based on their proximity to previously existing gaps. We divided the newly established gaps based on whether they were formed more or less than five meters (minimal diameter of a gap) from a previously existing gap. Another group was composed of gaps formed from previously existing gaps (overlap) that had decreased in size (more or less than 50% in area). Furthermore, gaps formed in the five meter buffer zone of previously existing gaps were classified in a separate group and divided based on whether previously existing gaps decreased in area more or less than 50%. The last group was made up of gaps that coalesced from two previous gaps.

## Results

### 1. Canopy gaps

**Figure 1:**
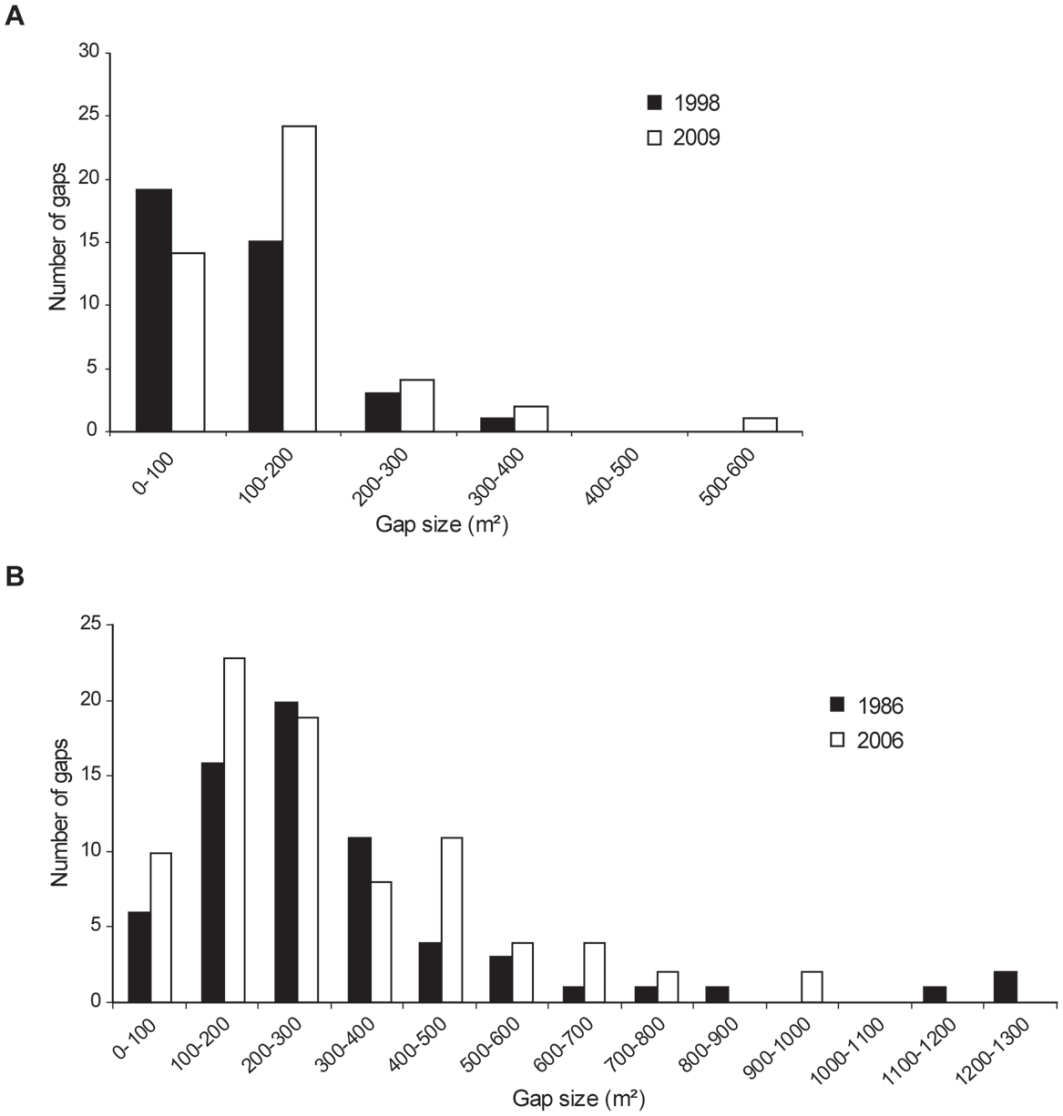
Gap size distribution in the Kopa (A) and Gorjanci (B) forest reserves.

Delineation of canopy gaps with a digital stereoplotter showed an increase in the gap fraction (proportion of the surface covered by gaps) in both reserves. In the Kopa reserve, the gap fraction increased from 3.2 to 4.5% (41%) during the eleven-year period. Average gap size increased from 118 to 141 m^2^ (Figure 1A).

Small gaps (≤200 m^2^) were most frequent while medium-sized gaps (200–600 m^2^) made up only a small number of gaps (Table 2; Figure 1A). Larger gaps (>600 m^2^) were not delineated in this period.

**Table 2:** Gap characteristics and gap dynamics processes in the Kopa forest reserve.

Year	1998	1998–2009				2009
		Gap dynamics processes				
Gap area (m^2^)		New gap formation	Gap expansion	Gap reduction	Gap closure	
Total	4483(319)	2080(148)	1498(107)	−579(41)	−1154(82)	6327(450)
No. of gaps	38(2.7)	22(1.6)	15(1.1)	11(0.8)	12(0.9)	45(3.2)
Gap size ≤200 m^2^	3464(247)	2080(148)	1220(86)	−361(26)	−1154(82)	4093(291)
No. of gaps	34(2.4)	22(1.5)	14(1.0)	8(0.6)	12(0.85)	38(2.7)
Gap size >200 m^2^	1019(73)	0(0)	278(20)	−218(15)	0(0)	2235(159)
No. of gaps	4(0.3)	0(0)	1(0.1)	3(0.2)	0(0)	7(0.5)

The numbers in parentheses are relative values calculated per forest reserve area.

The total change in gap area (169 m^2^ per year) was characterized by a large proportion of newly established and expanded gaps. These processes also involved the largest number of gaps. Gap closure and gap reduction represented a lower proportion (in area) but included 61% of all gaps captured in 1998. All gap dynamic processes were predominantly supported by small gaps. The annual proportion of the total area covered by new and closed gaps amounted to 0.15 and 0.08% of the whole forest area, respectively.

In the Gorjanci forest reserve, the gap fraction increased from 9.1 to 10.6% (17%) during the 20-year period. Average gap size decreased from 317 to 294 m^2^. The gap size distribution showed a predominance of small gaps (Figure 1A).

Gaps ranging from 100 to 300 m^2^ were most frequent, while gaps smaller than 100 m^2^ and gaps ranging from 300 to 500 m^2^ were less frequent. Larger gaps (>600 m^2^) were rare and reached up to 1200 m^2^ in size. Even though medium-sized gaps (>200 m^2^) were less frequent (Table 3) than small gaps (≤200 m^2^) in both measurements (47% in 1986 and 48% in 2006), they represented a dominant proportion in area (77% in 1986 and 74% in 2006).

**Table 3:** Gap characteristics and gap dynamics processes in the Gorjanci forest reserve.

Year	1986	1986–2006				2006
		Gap dynamics processes				
Gap area (m^2^)		New gap formation	Gap expansion	Gap reduction	Gap closure	
Total	20 903(910)	11 406(496)	4034(176)	−4812(209)	−7157(311)	24 374(1061)
No. of gaps	66(2.9)	482(2.1)	18(0.8)	17(0.7)	31(1.4)	83(3.6)
Gap size ≤200 m^2^	3052(133)	3475(151)	1837(80)	−1152(50)	−2067(90)	11310(492)
No. of gaps	23(1.0)	27(1.2)	6(0.3)	2(0.1)	17(0.7)	39(1.7)
Gap size > 200 m^2^	17850(777)	7931(345)	2197(96)	−4660(203)	−5090(222)	13064(569)
No. of gaps	43(1.9)	21(0.9)	12(0.5)	15(0.6)	14(0.6)	44(1.9)

The numbers in parentheses are relative values calculated per forest reserve area.

**Figure 2:**
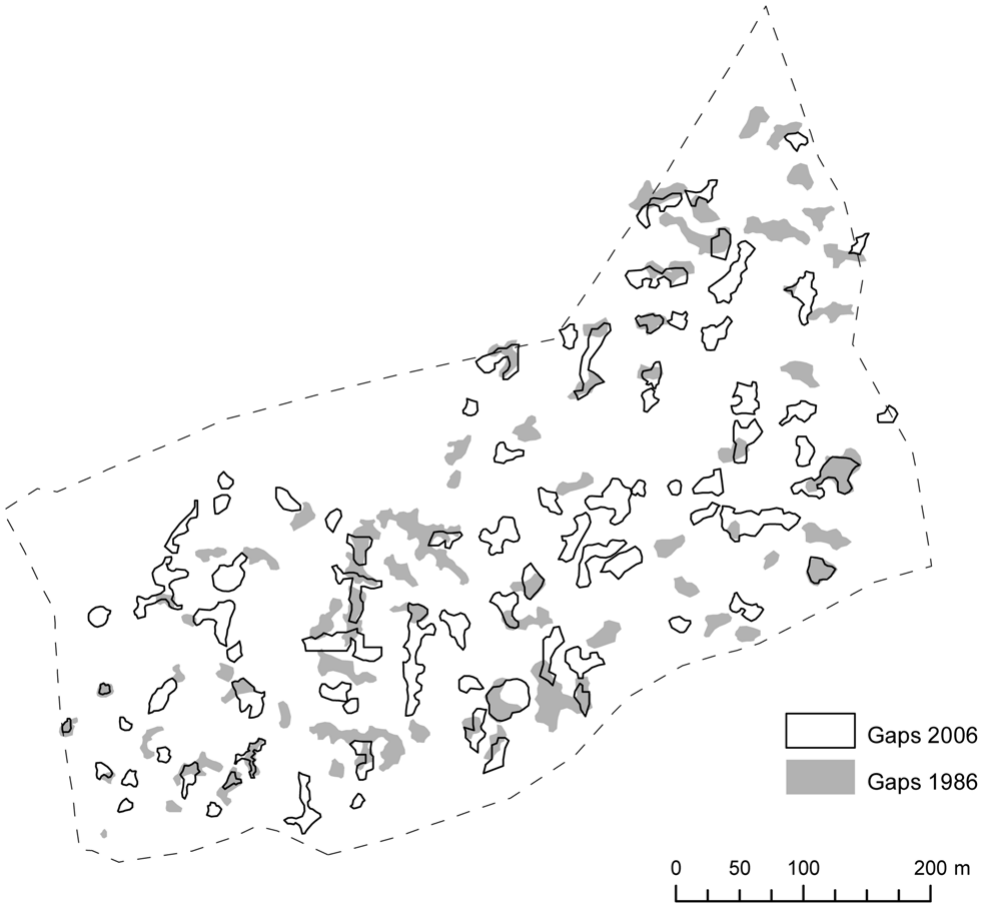
Gap dynamics in the Gorjanci forest reserve in the studied period (1986–2006).

**Figure 3:**
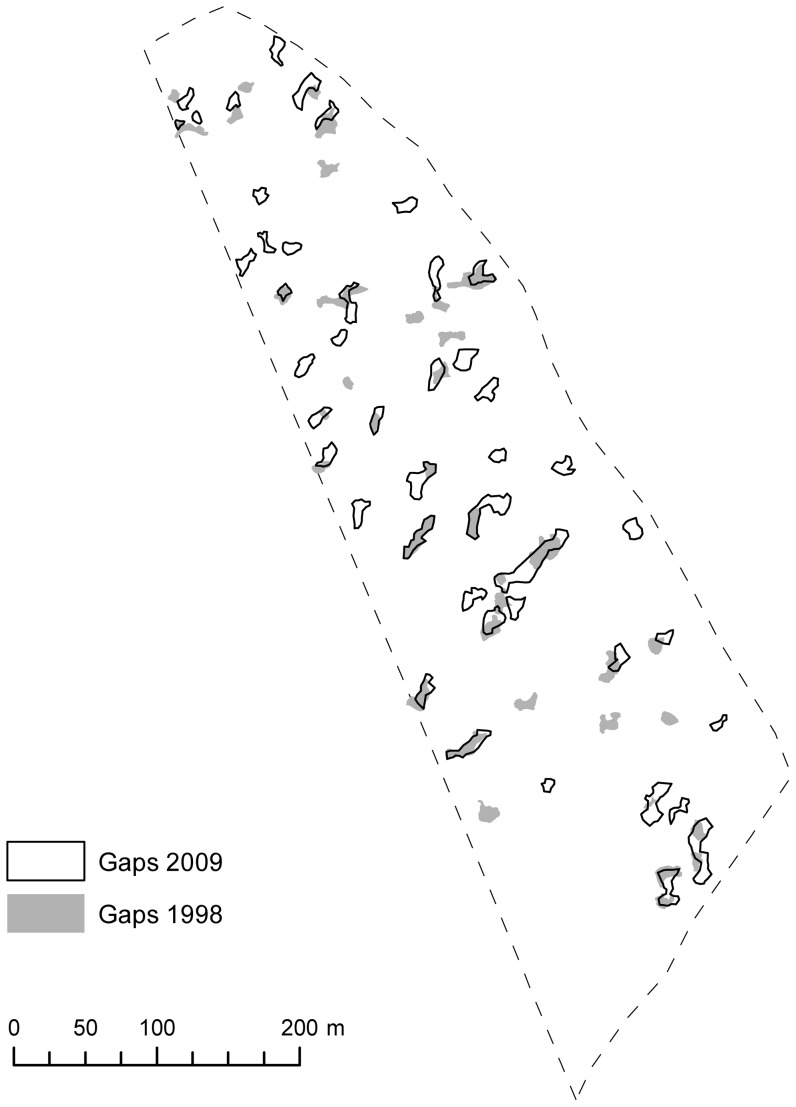
Gap dynamics in the Kopa forest reserve in the studied period (1998–2009).

The total change in gap area (174 m^2^ per year) was characterized by a large proportion (in area and in number) of newly established and closed gaps (Table 3, Figure 2). Gap expansion and gap reduction were similar (in area and in number). New gap establishment, gap expansion, and gap closure were dominated by small gaps (in number) and by medium-sized gaps (in area). In contrast, gap reduction was observed mostly in medium-sized gaps (in area and in number). The annual proportion of the total area covered by new and closed gaps amounted to 0.25 and 0.16%, respectively. The gap dynamics were comparable in the Kopa forest reserve (Table 2, Figure 3).

**Figure 4:**
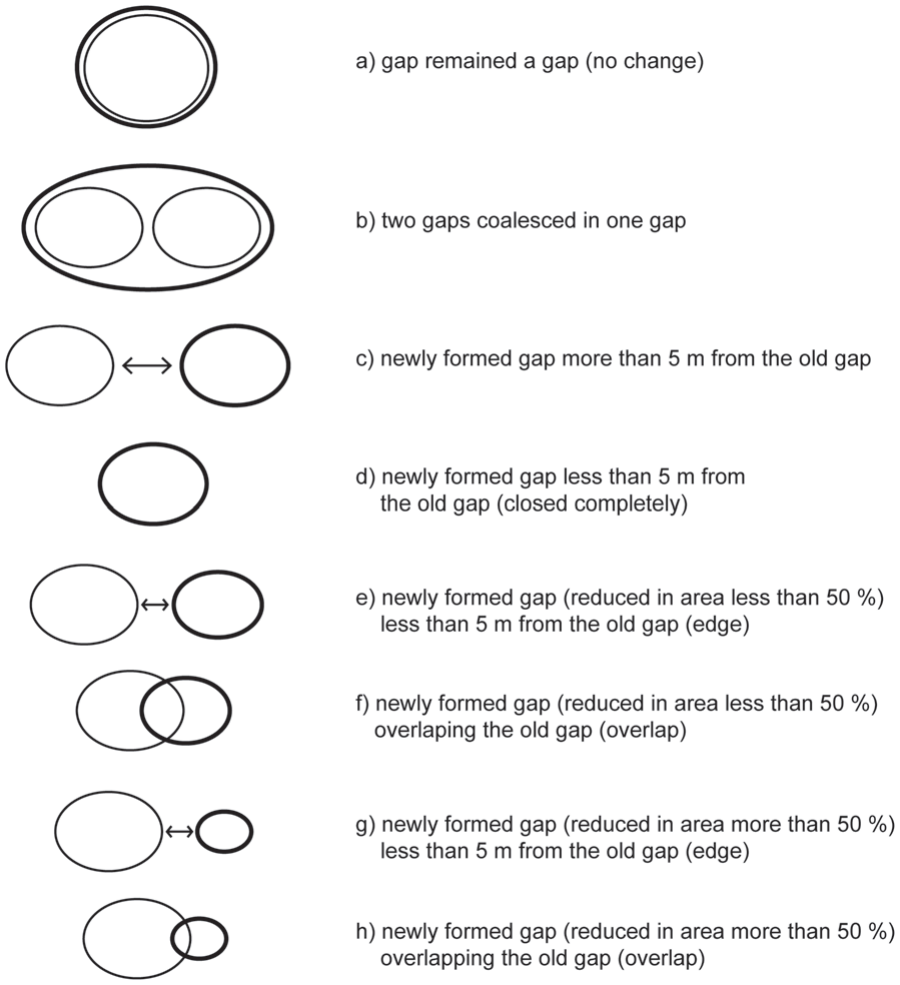
Gap formation processes (basic line represents gaps in the first measurement period, bold line represents gaps in the second measurement period).

A more detailed analysis of new gap formation (Figure 4) in the Kopa (Table 4) and Gorjanci (Table 5) forest reserves showed that 38% and 41% of gaps formed in the previously undisturbed stand (more than 5 m from the already existing gap) in Kopa and Gorjanci, respectively. In the five meter buffer zone of the previously existing and subsequently closed gap, one gap formed in Kopa and seven gaps in Gorjanci. Most of the gaps were reformed from previously existing gaps, thus partially overlapping the previous gap (47% in Kopa and 41% in Gorjanci). Only a few gaps formed in the five meter buffer zone of the older gaps (four in Gorjanci and one in Kopa). A few gaps (one in Gorjanci and two in Kopa) also coalesced from two smaller gaps. Only one gap in each reserve was enlarged with no reformation of the previous gap edge.

**Table 4:** Classification of newly established gaps in the Kopa forest reserve.

	1998 gap									
2009 gaps	Gap (1998) remained a gap (2009)	2 gaps (1998) coalesced in 1 gap (2009)	Newly formed gap (2009) >5 m from closed gap (1998)	Closed completely	Partial closure (<50% of area)		Partial closure (>50% of area)		No change	
	1 (2.2)	2 (4.4)	17 (38.0)							
	Formed on edge and overlap of 1998 gap			1 (2.2)	10 (22.2)		12 (26.7)		1 (2.2)	
	Edge	Overlap		1 (2.2)	0	10 (22.2)	1 (2.2)	11 (24.5)	0	1 (2.2)

The numbers in parentheses are percentages.

**Table 5:** Classification of newly established gaps in the Gorjanci forest reserve.

	1986 gap									
2006 gaps	Gap (1986) remained a gap (2006)	2 gaps (1986) coalesced in 1 gap (2006)	Newly formed gap (2006) >5 m from closed gap (1986)	Closed completely	Partial closure (<50% of area)		Partial closure (>50% of area)		No change	
	1 (1.2)	1 (1.2)	33 (40.7)							
	Formed on edge and overlap of 1986 gap			7 (8.6)	19 (23.5)		18 (22.2)		1 (1.2)	
	Edge	Overlap		7 (8.6)	2 (2.5)	17 (21)	2 (2.5)	16 (19.7)	0	1 (1.2)

The numbers in parentheses are percentages.

### 2. Forest structure

A complete inventory (2009) of the Gorjanci forest reserve showed that beech was the predominant tree species (97.8% of the total growing stock). Sycamore maple was present in minor proportion (2.2%) and other species, such as silver fir and European rowan (*Sorbus aucuparia* L.), were rare. In the Kopa forest reserve (complete inventory in 2006) beech, sycamore maple, and silver fir accounted for 91.7%, 5.8%, and 2.4% of the total growing stock, respectively, while other tree species such as European ash, small-leaved lime (*Tilia cordata* Mill.), wild cherry (*Prunus avium* L.), goat willow (*Salix caprea* L.), and European rowan, amounted to only 0.1% of the total growing stock.

The analysis of the sample plot data showed a relatively low live tree density in both reserves (Table 6). The variation between the plots was high (coefficient of variation – CV∼50%), indicating spatial variability of stem density. The basal area in the Gorjanci and Kopa forest reserves amounted to 41 and 39 m^2^ ha^−1^, respectively. The spatial variability of basal area was observed, with a coefficient of variation of 31% in the Kopa reserve and 27% in the Gorjanci reserve. Similarly, the amount of growing stock was relatively high, amounting to 595 m^3^ ha^−1^ in the Kopa reserve and 650 m^3^ ha^−1^ in the Gorjanci reserve. The analysis of the coarse woody debris (CWD) showed a large dead wood pool in these forest reserves. It comprised 19 and 21% of the total aboveground volume in the Kopa and Gorjanci reserves, respectively. It was spatially unevenly distributed, particularly in the Kopa reserve, where the coefficient of variation amounted to 108%.

**Table 6:** Main structural parameters of the Kopa and Gorjanci forest reserves. All parameters measured in the plots were scaled to one hectare.

	Kopa	Gorjanci
Number of sample plots	21	24
Living trees		
Stem density (N ha^−1^)Average	338 (581)	261
SD	171 (312)	131
CV (%)	51(54)	50
Basal area (m^2^ ha^−1^)		
Average	39 (40)	41
SD	12 (12)	11
CV (%)	31 (30)	27
Growing stock (m^3^ ha^−1^)		
Average	595 (597)	650
SD	194 (194)	181
CV (%)	33 (32)	28
CWD (m^3^ ha^−1^)		
Average	144 (144)	171
SD	155 (155)	70
CV (%)	108 (108)	41
CWD (%)	19 (19)	21

SD…standard deviation, CV…coefficient of variation, CWD…coarse woody debris.

The calculations were made for all trees with d.b.h.≥10 cm. The numbers in parentheses are calculations for all trees with d.b.h.≥5 cm.

**Figure 5:**
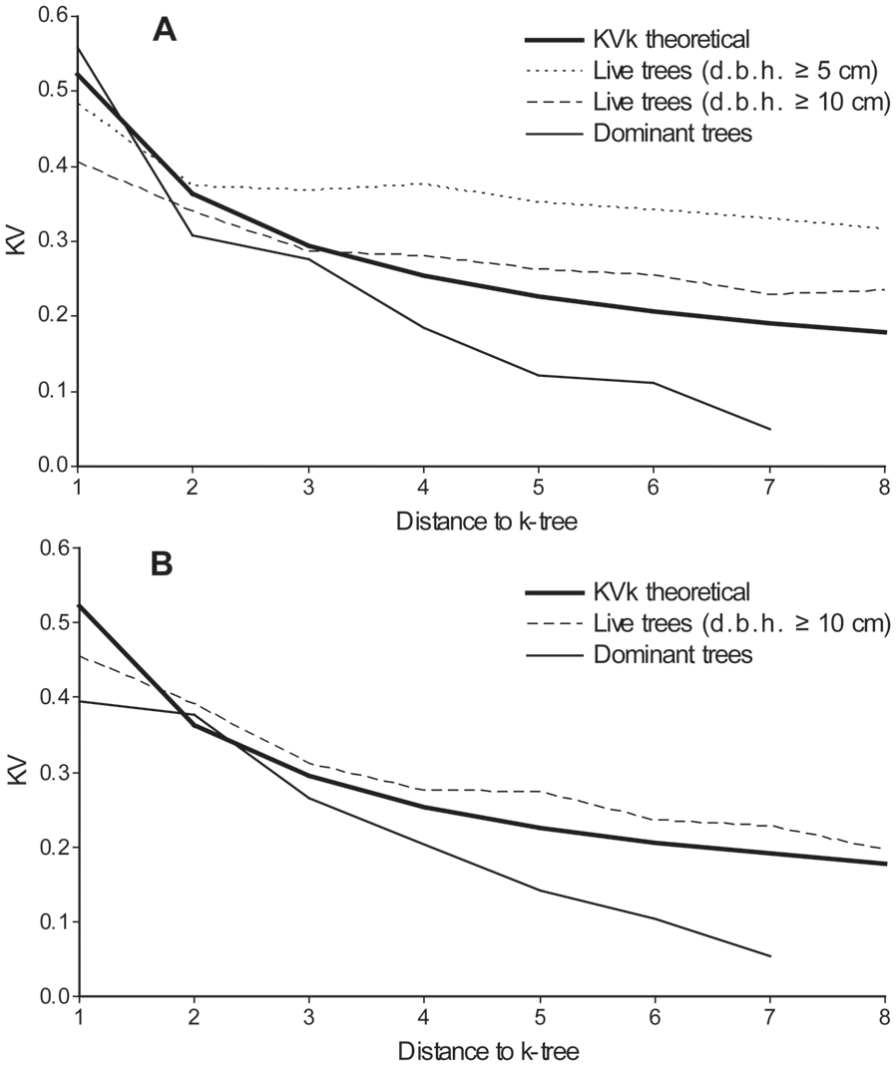
Spatial distribution of live trees in the Kopa (A) and Gorjanci (B) forest reserves. The bold line represents the theoretical (random) distribution of trees.

A strong tendency towards clustered distribution was observed for all trees with d.b.h.≥5 cm in the Kopa forest reserve. The tendency towards clustered distribution for all trees with d.b.h.≥10 cm was less pronounced in both reserves. The distribution of trees in the upper canopy layer (dominant trees) showed a distinct tendency towards regular distribution in both reserves (Figure 5).

**Figure 6:**
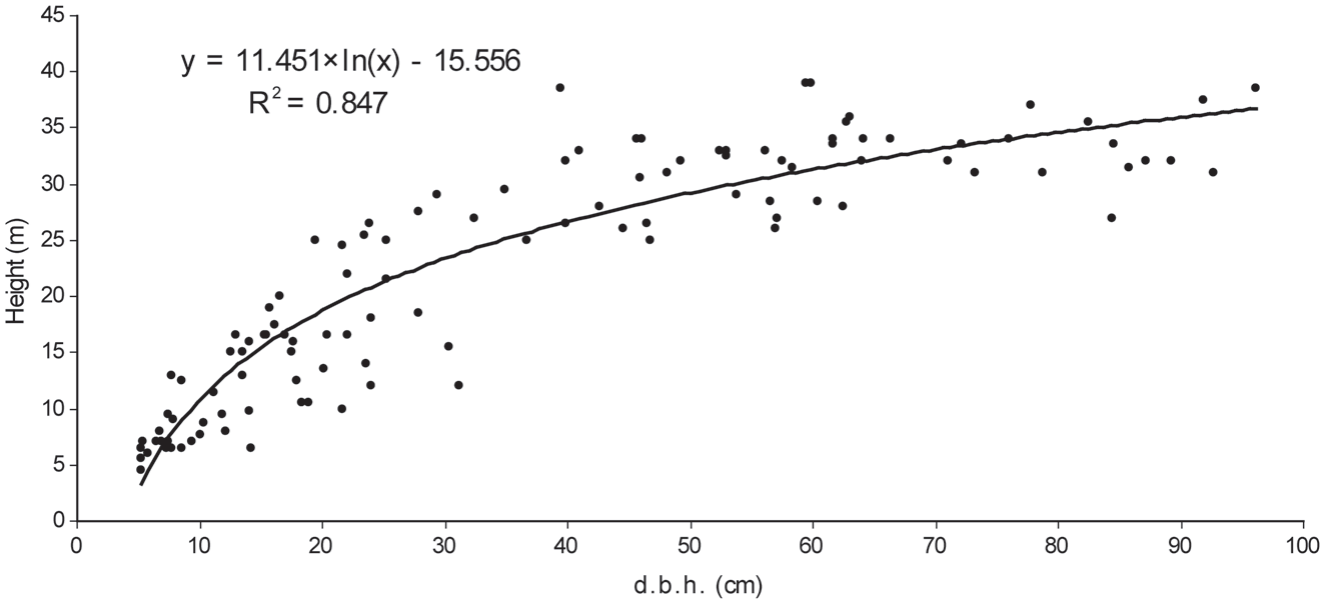
Height curve of beech in the Kopa and Gorjanci forest reserves. “d.b.h.” is diameter at breast height.

Figure 6 presents a height curve based on 113 measured beech tree heights in both reserves (72 in the Gorjanci reserve and 41 in the Kopa reserve). Tree heights reached up to 41 m. The upper canopy layer (25–40 m height) was constituted by trees of a wide d.b.h. range (from 20 to 110 cm). Trees belonging to the middle layer (10–25 m) ranged from 15 to 30 cm d.b.h.

**Figure 7:**
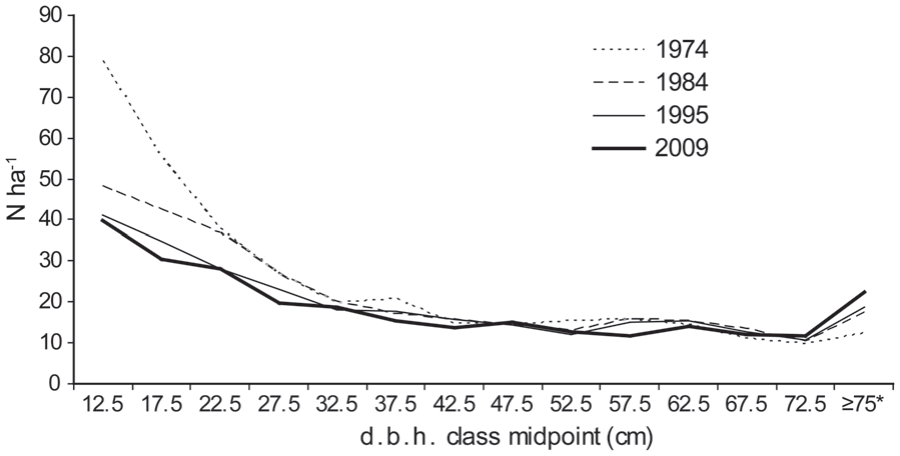
Diameter distribution (frequency of trees per 5-cm diameter classes) of all trees in the Gorjanci forest reserve. Note that all trees with d.b.h.≥75 cm were classified in one 5-cm d.b.h. class (*).

**Figure 8:**
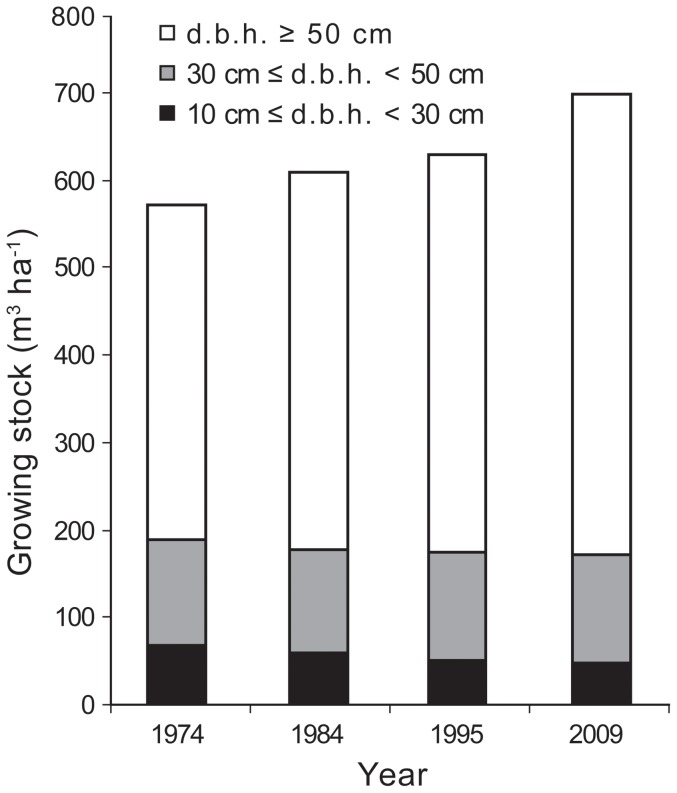
Distribution of growing stock in three d.b.h. classes in the Gorjanci forest reserve. “d.b.h.” is diameter at breast height.

A complete inventory of all trees (≥10 cm d.b.h.) in the Gorjanci forest reserve showed a decreasing distribution of trees per d.b.h. class (Figure 7). When trees up to 35 cm d.b.h. were examined, a decrease in number was observed over time. A substantial number of trees up to 25 cm d.b.h. were measured in the first measurement (1974). The number of trees with 30 cm≤d.b.h.<75 cm remained relatively constant over the entire measurement period (1974–2009). The number of trees above 75 cm d.b.h. increased over time. This trend was partially masked by the fact that all trees above 75 cm d.b.h. were classified in the same size class (d.b.h.≥75 cm). Growing stock development in the Gorjanci forest reserve showed an increase in the measurement period (Figure 8). This increase was solely due to an increase within the largest-diameter class (d.b.h.≥50 cm).

**Figure 9:**
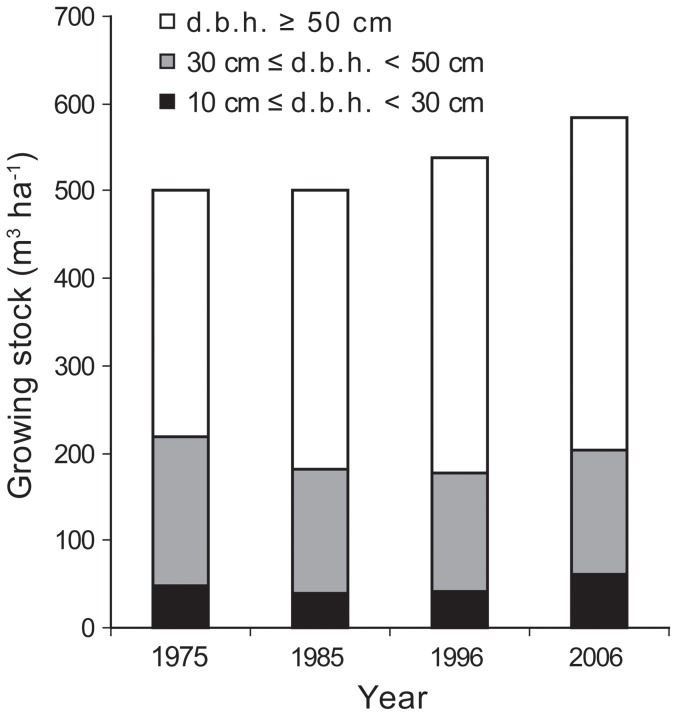
Distribution of growing stock in three d.b.h. classes in the Kopa forest reserve.

Similarly, a continuous increase in total growing stock was observed in the Kopa forest reserve from 1985 to 2006 (Figure 9). The increase in growing stock was most pronounced in the largest-diameter class (d.b.h.≥50 cm). The volume of growing stock in the small-diameter class (10 cm≤d.b.h.<30 cm) increased in the last decade measured, while in the previous decades the volume of growing stock was similar. The volume of growing stock in the medium-diameter class (30 cm≤d.b.h.<50 cm) decreased in the first decade and remained at the same level in the last three measurements (21-year period).

## Discussion

Gap size analysis in the Kopa reserve showed that small gaps (≤200 m^2^) are the dominant driving force of forest dynamics. Medium-sized gaps were rare, and large gaps were not present. The gap fraction increased by 41%, but still remained under 5% of the total area studied. The gap fraction is as low as in some other Southern European beech reserves [11], [19]. In the Kopa reserve, medium-sized gaps (>200 m^2^) comprised 23% (1998) and 35% (2009) of the total gap area, which is less than what was observed in a similar beech dominated old-growth forests in Slovenia [19]. New gap formation and expansion of small gaps seem to be the main driving forces behind stand dynamics and are mitigated by gap closure. Small gaps are most likely closed by the lateral growth of tree crowns [2], [43]. Average gap size in the Kopa reserve was 141 m^2^ in 2009 and was less than half the average gap size in the Gorjanci reserve in 2006 (297 m^2^). In the Gorjanci reserve, the gap fraction was higher (10.6% in 2006), which is comparable with other beech forest reserves [10], [26]. Medium-sized gaps were more frequent here (more than 50% in number) and they constituted three quarters of the total gap area. Large gaps (>1000 m^2^) were very rare, even though there have been some reports of large disturbances in beech forest reserves in Slovenia [44], France [10], and Romania [17]. Most of the changes in gap area resulting from gap formation and gap closure occurred in medium-sized gaps, which did not exceed 1200 m^2^. Annual rates of gap closure and new gap formation are not as high as those reported in some other forest types [45], but are substantially higher than those found in some beech reserves in Europe [26]. Kenderes et al. [26] presented annual rates of the total area covered by new gaps between 0.05 and 0.09% and annual rates of the total area covered by closed gaps between 0.01 and 0.03%. Our results suggest that gap disturbance regime governed by small gaps can exhibit relatively large changes in gap area over short periods of time. The process of new gap formation is related to the presence of older gaps since a high proportion of the gaps (46–51%) were formed in the vicinity (less than five meters) of the older gaps. This proportion is larger to that of the gaps formed in the previously undisturbed canopy (38–41%). It seems that the process of new gap formation is more frequent in the vicinity of the previously existing gap since the gap area is 10 to 20 times smaller than the area of the undisturbed canopy.

A similar conclusion can be drawn from the variability of stand characteristics estimated in the sampling plots. The variation in basal area and volume per hectare in our study, measured by the coefficient of variation, was between the 20% found in virgin beech forest in Ukraine [13] and the 38% found in beech dominated natural forests in the Czech Republic [46], whereas the variation in tree number was 10 to 13% greater in our study. The high standard deviation (131 to 171) and coefficient of variation (∼50%) suggest that these forests are structurally (d.b.h. structure) heterogeneous over small spatial scales. Such high variability has only been detected in Italian [47] and Czech [46] non-managed beech stands, but not in Slovakian ones [48]. Mean tree densities in the Kopa (338 per hectare) and Gorjanci (261 per hectare) forest reserves are comparable with other beech dominated old-growth forests in Europe [6], [11], [13], [14], [15], [19], [16]. Basal area comprises around 40 m^2^ ha^−1^ and varies up to 30%, indicating a relatively stable spatial distribution. Higher values (up to 52 m^2^ ha^−1^) have been observed in some comparable forests although lower values have been determined in stands with a shorter history of non-management [49].

The average growing stock of live trees was 595–650 m^3^ ha^−1^ and had similar variability as did basal area (28–33%). Much higher values (up to 1268 m^3^ ha^−1^) have been detected in some reserves in Slovakia, Ukraine, and Albania. We observed a high proportion of CWD (19–21% of total growing stock) in the Kopa and Gorjanci reserves. The amount of CWD in both reserves is high compared with similar European beech old-growth forests [50]. CWD was distributed very unevenly in the forest (CV up to 108%), indicating very localized, small disturbance events in the otherwise undisturbed background canopy layer.

Since beech is a shade-tolerant tree species, groups of seedlings can establish under small canopy openings and persist for longer periods of time, thus sustaining themselves only on scarce, moving sun flecks [2], even after canopy gaps are closed. When the spatial distribution of the dominant (upper canopy layer) trees was observed, a uniform distribution was evident. This pattern was also observed in some other European old-growth forests [51]. This may be one of the reasons for the ‘cathedral like’ appearance of pure beech forests.

Tree height analysis showed that the middle layer (11–22 m) was very poorly represented. This could be explained by the fact that beech grows through the pole-sized and intermediate-diameter classes very quickly [6], [13]. This may also explain the ‘cathedral-like’ appearance of beech stands [18]. Successful trees reach the canopy layer (22 m), thus forming a wide diameter distribution of upper storey trees (20–110 cm d.b.h.), similar to other old-growth European beech reserves [13].

The time series of tree diameter distribution in the Gorjanci forest reserve showed a constant decrease in the number of trees smaller than 25 cm in d.b.h. in the measurement period. It is likely that in the preceding period there had been a higher intensity disturbance event, which resulted in favorable light conditions for successful seedling recruitment. Such events have also been detected in beech dominated old-growth forests in Slovakia [6]. It could also be the result of unrecorded felling after World War Two, when a forest road was constructed in the vicinity of the reserve. Because of the probable constant closure of the canopy, we did not find any increase in tree frequency in the larger d.b.h. classes in later periods. This can be attributed to the high mortality among beech saplings under closed canopy. The development of growing stock in the Kopa reserve also indicates a relatively stable rate of increase over the last three measurement periods (1985–2006). The proportion of trees in the growing stock with d.b.h. larger than 50 cm increased, indicating ageing of this forest reserve. An increase in growing stock was also observed in the Gorjanci forest reserve.

Structural characteristics of the forests in our study indicate diverse diameter structure and a less diverse height structure. Consequently, we conclude that these stands are not even-sized (uniform) as proposed by some authors [2], but rather unevenly structured. This is due to the fact that the disturbance regime is characterized by small-scale, low-intensity disturbances that are variable in space and time. These findings largely support the third conceptual model presented in the introduction, although the lack of trees in the middle layer recorded in this study may also lend support to the second model. Because large-scale, high-intensity disturbances are rare, which limits the possibility of synchronous recruitment over larger areas, the first conceptual model is not supported. Yet, it cannot be excluded due to the small size of both reserves and short time interval of measurements. For a better understanding of the disturbance regime in beech dominated old-growth forests, we propose more meta-analyses of similar studies in Europe, as well as analysis of larger areas and the application of dendroecological methods. This would provide an important insight into the disturbance history and age structure of these stands as well as a better understanding of past human land-use practices.
